# Effects of *Ginkgo biloba* on Early Decompression after Spinal Cord Injury

**DOI:** 10.1155/2020/6958246

**Published:** 2020-06-03

**Authors:** Xing Guo, Xiaotie Wang, Jin Dong, Wei Lv, Shandou Zhao, Liangliang Jin, Jiangtao Guo, Mingfeng Wang, Chaojun Cai, Jie Sun, Yan Zhou, Qian Zhang, Xiaoli Chen, Jinyan Yang, Ruili Ji, Xiaoyan Xin, Min Li, Yongsong Li, Jin Fan

**Affiliations:** ^1^Department of Orthopedics, General Hospital of Jincheng Anthracite Mining Group Co., Ltd., Jincheng, China; ^2^Department of Intensive Care Unit, Zezhou People's Hospital, Jincheng, China

## Abstract

Spinal cord injury (SCI) is a severe trauma of the central nervous system characterized by high disability and high mortality. Clinical progress has been achieved in understanding the pathological mechanism of SCI and its early treatment, but the results are unsatisfactory. In China, increasing attention has been paid to the role of traditional Chinese medicine in the treatment of SCI. In particular, extracts from the leaves of *Ginkgo biloba* (maidenhair tree), which have been reported to exert anti-inflammatory and antioxidant properties and repair a variety of active cellular damage, have been applied therapeutically for centuries. In this study, we established a rat SCI model to investigate the effects of *Ginkgo biloba* leaves on decompression at different stages of SCI. The application of *Ginkgo biloba* leaves during the decompression of SCI at different time points, the neurological recovery of SCI, and the underlying molecular mechanism were explored. The findings provide reliable experimental data that reveal the mechanism of GBI (*Ginkgo biloba* injection) in the clinical treatment of SCI.

## 1. Introduction

In recent years, the incidence of spinal cord injury (SCI) has been increasing. Progress has been made in understanding the pathological mechanism of SCI and its early treatment, but the results have been unsatisfactory [1, 2]. At present, it is believed that hemorrhage caused by secondary injury, which is characterized by edema, apoptosis, and immune inflammatory cascade, plays an important role in the destruction of spinal nerve tissue and affects the prognosis of SCI, with prominent effect on inflammatory response [3, 4]. A large amount of class III clinical data have demonstrated that surgical decompression is a feasible treatment of acute SCI. Clinical and basic experimental studies have also shown that early surgical decompression after SCI exerts protective effects on the injured spinal axonal cord, reducing the area of SCI and promoting the recovery of hind limb functions [5, 6].

The development of traditional Chinese medicine has led to increasing attention being focused on its application in the treatment of SCI in China. The extracts from the leaves of *Ginkgo biloba* (maidenhair tree; see Graphical Abstract) reportedly exert anti-inflammatory, antioxidant, and neuroprotective properties and can repair a variety of active cellular damage. These extracts have been used therapeutically for centuries [7] in the clinical treatment of disorders associated with cerebral circulation and peripheral blood circulation [8–10]. The effects of ginkgo may be induced by single active ingredients found in the extracts or by their combined action. Mechirova and Domoráková found that the *Ginkgo biloba* extract Tanakan effectively eliminated free radicals generated during lumbar ischemia and reperfusion in rabbits and reduced reperfusion injury [11]. Meanwhile, Cheng et al. reported that *Ginkgo biloba* extract improved neuronal cell damage after spinal cord ischemia and reperfusion via the mitochondrial pathway [12]. Song et al. revealed the protective effects of *Ginkgo biloba* extract Ginkgolide B against acute SCI in rats, which may be related to the JAK/STAT signaling pathway [13]. Current reports have focused on the effects of *Ginkgo biloba* extracts on neuronal apoptosis and their neuroprotective effects after SCI [14, 15]. However, whether *Ginkgo biloba* extracts inhibit spinal cord inflammation after secondary injury while simultaneously exerting neuroprotection after early decompression has not yet been reported.

Based on the above theory, we hypothesized that *Ginkgo biloba* leaves can alleviate inflammatory reaction after secondary SCI and protect functional cells, such as neurons and oligodendrocytes, thereby promoting the repair of SCI. This study aims to provide a reference for the application of traditional Chinese medicine in clinical SCI.

## 2. Materials and Methods

### 2.1. Animals

Sprague-Dawley rats weighing 200–210 g (6-7 weeks of age) were purchased from Liaoning Changsheng Bio. Co., Ltd. with approval from the ethics committee and divided into two groups (30 for control, 150 for SCI). All animal experiments were performed based on the Guidelines for Animal Care and Use of the Model Animal Research Institute at Wuhan Myhalic Biotechnology Co., Ltd. The Institutional Review Board confirms that the scheme of this project was properly designed, the number of animals required was limited to the minimum, the investigators were qualified to carry out the proposed project, and all animals were handled with sufficient care and protection.

### 2.2. Modeling and Treatment with *Ginkgo biloba*

#### 2.2.1. Construction of SCI Model

Sprague-Dawley rats subjected to SCI modeling were anesthetized intraperitoneally using 1% pentobarbital sodium at 30–40 mg/kg. An acute SCI model of continuous static compression was established in rats by cyclization [16]. Throughout the study, the experimental rats were paralyzed immediately. Rats subjected to SCI were randomly divided into four experimental groups and treated as follows: simple decompression 8 h after SCI without drug intervention, simple decompression 8 h after SCI with *Ginkgo biloba* injection (GBI), simple decompression 48 h after SCI without drug intervention, and simple decompression 48 h after SCI with GBI. All rats were sacrificed 3 and 60 days after injury via administration of an overdose of sodium pentobarbital.

#### 2.2.2. *Ginkgo biloba* Intervention

*Ginkgo biloba* (Chi Sheng Pharma & Biotech Co., Ltd., Taiwan) was dissolved in 0.5% sodium carboxymethyl cellulose solution. GBI was performed via daily intraperitoneal injection (4 mg/kg of body weight) for two weeks after SCI. Meanwhile, control and nondrug-treated rats received the same volumes of 0.5% carboxymethyl cellulose solution at the same times.

### 2.3. Basso–Beattie–Bresnahan Score

Neurological assessments were performed daily after SCI. The open-field test assessed the rats' locomotion, weight support, and coordination, and the results were evaluated using the Basso, Beattie, and Bresnahan (BBB) scale (range from 1 to 21), as previously described (Basso et al., 1995). A score of 0 represented no spontaneous movement, whereas a score of 21 represented complete mobility. The BBB curve was constructed using data from selected time points.

### 2.4. Tissue Preparation and Histological Examination

After sacrifice, half of the spinal cord tissues were obtained and stored at −80°C, and the remaining half of the samples were fixed in 4% paraformaldehyde. The nerve myelin sheath, morphology, and number of neurons were observed by Luxol fast blue (LFB) (servicebio.cn) and Nissl staining. The number of nerve fibers was examined by NF-200 staining (Proteintech, 1 : 200) and astrocytes and glial scars were assessed by glial fibrillary acidic protein (GFAP) staining (Proteintech, 60190-I-IG, 1 : 200). All images were acquired using a microscope (Leica, DM1000) and the Leica Application Suite image system was used to collect and analyze relevant parts of the sample.

### 2.5. Terminal Deoxynucleotidyl Transferase dUTP Nick End Labeling (TUNEL)

Paraffin-embedded tissue specimens were sliced and underwent conventional dewaxing and hydration. The sections were placed into 3% hydrogen peroxide methanol for 15 min, and each sample was incubated with 100 *μ*l of protease K (20 *μ*g/ml) for 20 min. After two washes with phosphate-buffered saline for 3 min each, the samples were incubated with a TUNEL apoptosis detection kit (11684817910, Roche) according to the manufacturer's instruction. The sections were sealed, dried, and observed using an optical microscope (CX41, OLYMPUS).

### 2.6. Macrophage Infiltration

Flow cytometry (BECKMAN, CytoFLEX S) was performed to detect the infiltration of inflammatory cells in spinal cord tissues, including neutrophils (CD11b-FITC, eBioscience, Rockland Immunochemicals Inc.; transcription factor eGr-1, eBioscience, Rockland Immunochemicals Inc.) and peripheral mononuclear macrophages (CD45-FITC, eBioscience, Rockland Immunochemicals Inc.). Immunohistochemical staining of F4/80 was carried out to assess the infiltration of macrophages (Thermo Fisher, 1 : 200). All samples were obtained three days after SCI.

### 2.7. Western Blot

Dorsal white matter strips were isolated and stored at −80°C for the detection of inflammatory proteins and cleaved caspase-3 (abcam, ab2302, 1 : 1000), Bax (Bioswamp, PAB30861, 1 : 1000) and Bcl-2 (Bioswamp, PAB44408, 1 : 1000) in the spinal cord. The spinal cord tissue was cut into small pieces, and radioimmunoprecipitation assay buffer containing was protease and phosphatase inhibitors added at a ratio of 150–250 *μ*L of lysate per 20 mg of tissue. The tissue was then homogenized until complete lysis was achieved. The lysed sample was centrifuged at 12,000 ×*g* for 1 minute at 4°C, and the supernatant was quantified and stored at −80°C. The expression of the following factors was detected: Gro-*α* (abcam, ab86436, 1 : 2000), a chemotactic activity for neutrophils; monocyte chemoattractant protein 1 (MCP1, abcam, ab25124, 1 : 2000); macrophage inflammatory protein 2 (MIP2, abcam, ab25130, 1 : 2000), MIP1 (abcam, ab25128, 1 : 2000), and MIP1b (abcam, ab25129, 1 : 2000), produced by activated monocytes and neutrophils; tumor necrosis factor-*α* (TNF-*α*, abcam, ab66579, 1 : 2000); interleukin 1b (IL-1b, Bioswamp, PAB30679, 1 : 2000), IL6 (abcam, ab208113, 1 : 1000), inducible nitric oxide synthase (iNOS, abcam, ab3523, 1 : 500), and cyclooxygenase-2 (COX-2, abcam, ab52237, 1 : 1000). Secondary antibody incubation was performed with horseradish peroxidase-conjugated goat anti-rabbit IgG (Bioswamp, PAB160011, 1 : 20000). GAPDH (Bioswamp, PAB36264, 1 : 2000) serves as an internal reference.

### 2.8. Statistical Analysis

All data were expressed as the mean ± SD. Data analysis was performed using GraphPad 7.0 software. Repeated-measures analysis of one-way analysis of variance was performed to compare the inflammatory infiltrate and proteins among groups. Differences were considered statistically significant at *P* < 0.001.

## 3. Results

### 3.1. Effect of *Ginkgo biloba* on Locomotor Activity

**Figure 1:**
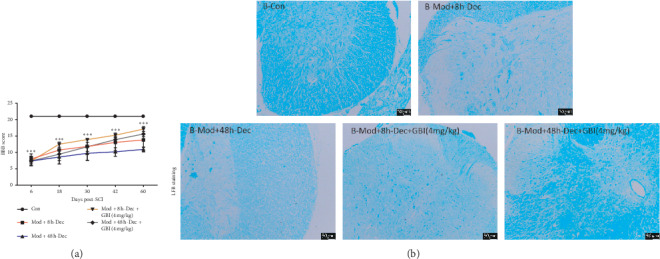
BBB scores and LFB staining before and after injury. (a) BBB scores. Data are expressed as the mean ± SD (*n* = 15). ^*∗*^*P* < 0.0001 vs. control. SCI: spinal cord injury; d: day(s); BBB: Basso, Beattie, and Bresnahan. (b) Luxol fast blue staining of spinal cord sections at 60 days after SCI. Scale bars: 50 *μ*m.

All rats initially had a BBB score of 21, and rats subjected to SCI showed a BBB score of 0–2 on the first day, which reflected total hindlimb paralysis. At 1 and 3 days after injury, the movement of the injured rats remained severely impaired, but decompression treatment at 8 h and 48 h after SCI resulted in significant improvements in mobility. Compared to decompression only, injured rats subjected to GBI (4 mg/kg/d) after 8 h after SCI decompression showed greater locomotor activity that continued throughout the experimental period (*P* < 0.0001; Figure 1(a)).

### 3.2. Effect of GBI Treatment on Demyelination

To observe the difference in the area of myelin sheath in rats with SCI after decompression, LFB staining of myelin tissue was performed in all rats at 60 days. The pathological results shown in Figure 1(b) demonstrate that combined decompression and GBI induced significantly larger areas of myelin sheath than those induced by decompression only. These results indicate that decompression could reduce myelin sheath loss after injury, and GBI further strengthened the protective effect of decompression.

### 3.3. Effect of GBI on Nerve Fibers, Neurons, and Astrocytes in Spinal Cord Tissue

**Figure 2:**
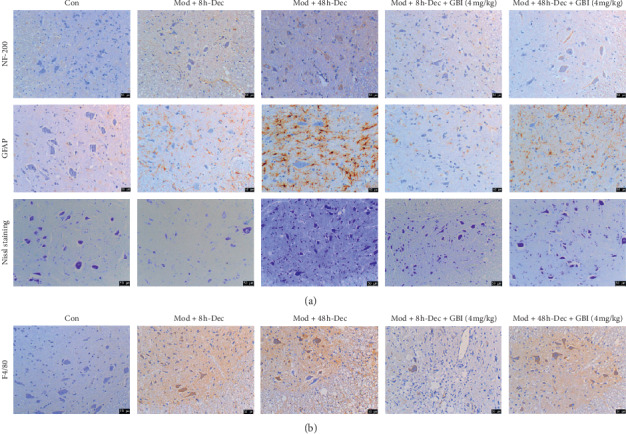
Nerve fibers, astrocytes, neuron cells, and macrophage infiltration in spinal cord tissue. (a) Immunohistochemical staining of NF-200 (nerve fibers), GFAP (astrocytes); and neurons (Nissl) in spinal cord tissue. (b) Immunohistochemical staining of F4/80 (macrophage infiltration), scale bars: 50 *μ*m. All immunohistochemistry experiments were performed 60 days after SCI.

Experimental rats were sacrificed 60 days after being subjected to SCI. Immunohistochemical staining was performed in spinal cord tissue to observe the characteristics of nerve cells after GBI and/or decompression (Figure 2(a)). The combination of post-SCI decompression and GBI after decompression significantly downregulated the expression of NF-200. On the other hand, GFAP, a marker of astrocyte damage, was significantly upregulated after SCI compared with the control group, but decompression at 8 h after SCI effectively downregulated its expression. GBI after decompression further attenuated the expression of GFAP, suggesting that the combination of decompression at 8 h after SCI with GBI ameliorated astrocyte damage and scar formation. Nissl staining also showed that decompression at 8 h after SCI in conjunction with GBI was more effective than simple decompression in repairing neuronal cell damage. These results were complemented by the immunohistochemical staining of F4/80 for macrophage infiltration in injured tissues (Figure 2(b)). As shown in Figure 2(b), compared with the Mod + 8 h-Dec group, the macrophage infiltration in the Mod + 48 h-Dec group is more severe; after GBI treatment on the basis of decompression, the proportion of inflammatory infiltration in the Mod + 8 h-Dec + GBI group was lower than that in the Mod + 8 h-Dec + GBI group, suggesting that GBI combined with decompression treatment can reduce the inflammation infiltration of spinal cord tissue, and the earlier the decompression time, the better the effect of GBI.

### 3.4. Effect of *Ginkgo biloba* on Apoptosis in the Spinal Cord

**Figure 3:**
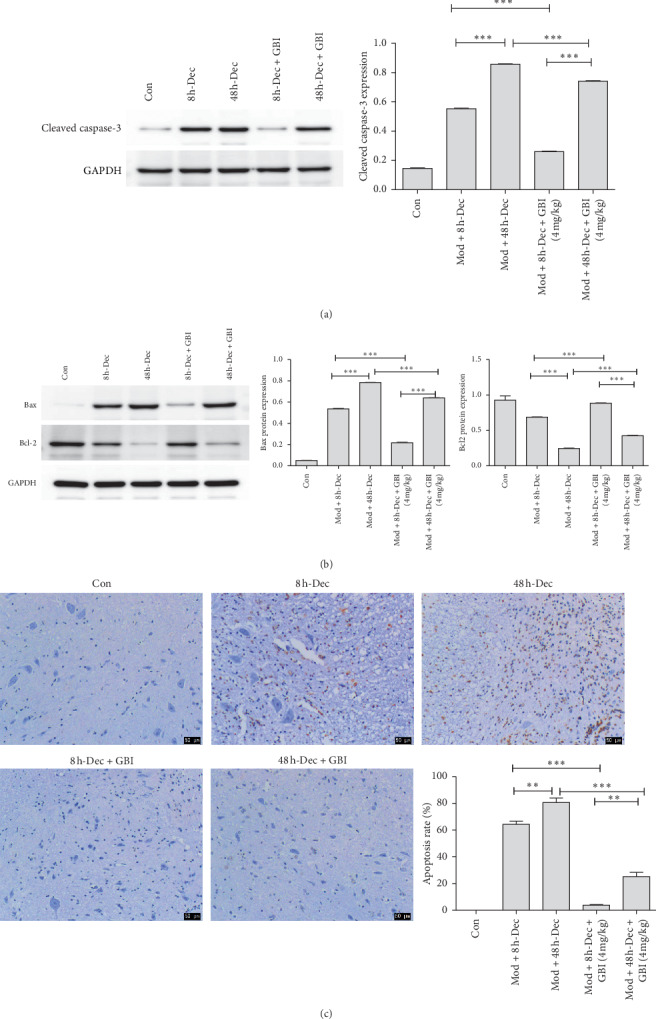
Expression of cleaved caspase-3 in spinal cord tissue. (a, b) Western blot and corresponding quantification of cleaved caspase-3, bax, and bcl-2 expression. GAPDH was used as a loading control. (c) Cell apoptosis was detected by TUNEL staining, scale bars: 50 *μ*m. Data are expressed as the mean ± SD (*n* = 15; one-way analysis of variance and *t*-test). ^*∗*^*P* < 0.05, ^*∗∗*^*P* < 0.01, ^*∗∗∗*^*P* < 0.001 vs. control.

After establishment of the SCI model, apoptosis in the spinal cord tissue was evaluated. Spinal cord tissue was extracted from rats treated with post-SCI decompression and GBI, and the expression of apoptotic proteins was detected by western blot. The protein expression of cleaved caspase-3 and Bax was significantly increase in 8 h and 48 h compared to the control, and GBI accentuated the effect of decompression in both cases (*P* < 0.001) (Figures 3(a) and 3(b)). Bcl-2 expression in Mod + 48 h-Dec group was significantly decreased compared to the Mod + 8 h-Dec group (*P* < 0.001), and GBI treatment promotes Bcl-2 expression. (Figure 3(b)). We also used TUNEL staining to detect the apoptotic rate, and the results support the above conclusions (Figure 3(c)).

### 3.5. Effect of *Ginkgo biloba* on Post-SCI Inflammatory Response

**Figure 4:**
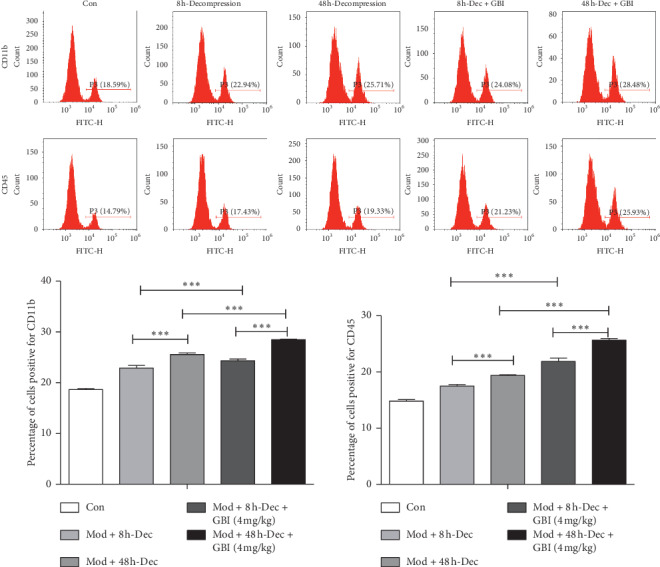
Inflammatory (macrophage) infiltration. Flow cytometry of the percentage of cells positive for surface markers of glial cells (CD11b and CD45). The experiments were performed 3 days after SCI.

**Figure 5:**
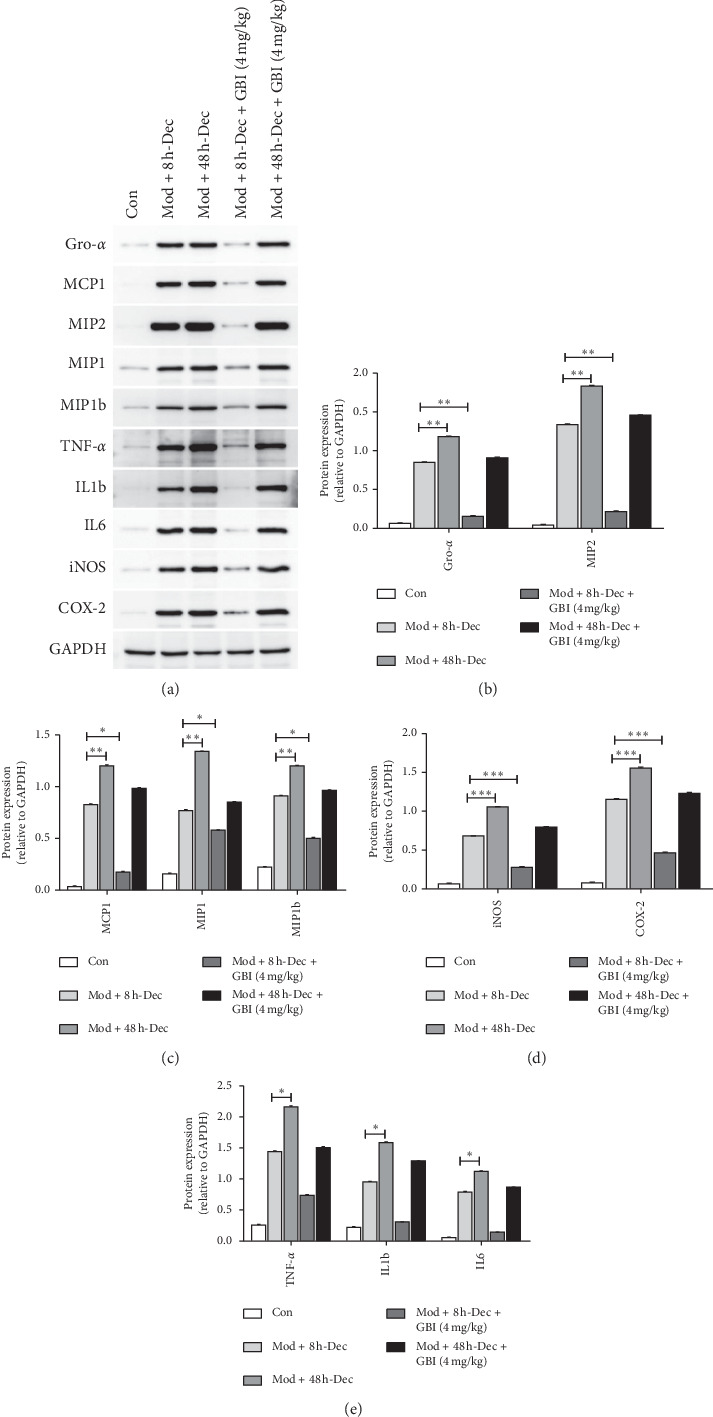
Production of inflammatory factors. (a) Western blot of the expression of inflammatory proteins. (b–e) Quantification of the proteins in (a). Densitometric analysis was used to estimate the intensity of bands. GAPDH was used as a loading control. Data are expressed as the mean ± SD (*n* = 15; one-way analysis of variance and Tukey's post hoc test). ^*∗*^*P* < 0.05; ^*∗∗*^*P* < 0.01; ^*∗∗∗*^*P* < 0.001 vs. control.

After SCI, a large number of activated glial cells and macrophages infiltrate the injured site. This results in the secretion of inflammatory proteins, which in turn aggravates tissue damage. To further study the activation of microglia after SCI, CD11b and CD45, which are markers of activated glial cells, were detected by flow cytometry 3 days after rats were subjected to SCI (Figure 4). The results showed that decompression at 8 h after SCI led to lower proportion of microglia than that at 48 h after SCI. On the basis of decompression treatment, after GBI, microglia cells were further suppressed, showing lower proportion with decompression at 8 h than at 48 after SCI in combination with GBI. Western blot was also carried out to examine the expression of inflammatory factors produced by glial cells and macrophages (Figure 5), 60 days after rats were subjected to SCI. Results showed that the expression of Gro-*α*, MIP2, MCP1, MIP1, MIP1b, iNOS, COX-2, TNF-*α*, IL1b, and IL6 in the mod + 48 h-Dec group was significantly higher than that in the mod + 8 h-Dec group (*P* < 0.05, *P* < 0.01, *P* < 0.001). However, after GBI treatment, the expression of the above inflammatory factors decreased significantly, which further confirmed that GBI treatment could inhibit the inflammatory response of spiral core tissue.

## 4. Discussion

SCI is a severe trauma of the central nervous system that affects a large proportion of young adults. It has become a global medical and social problem that results in burden such as disability, high cost of treatment, and low survival [1]. *Ginkgo biloba* is a product of lipid peroxidation that exerts potential protective function in spinal cord myelin, glial, and neuronal membranes and other cellular elements. Studies have pointed out that the *Ginkgo biloba* extract EGb761 has protective effects against brain death-induced kidney injury, and this effect may be related to SAPK and JAK-STAT signaling [17]. Clinical data have suggested that surgical decompression is a feasible treatment scheme for acute SCI, but whether *Ginkgo biloba* can inhibit the inflammation of the spinal cord after secondary injury with simultaneous neuroprotection after early decompression has not been reported.

Myelin integrity is an important factor in maintaining the physiological function of the central nervous system. The preservation and regeneration of myelin integrity after SCI plays a key role in the recovery of spinal cord function [18, 19]. Acute disturbance of nodal organization after SCI exposes the axon and triggers conduction block in the absence of overt demyelination. Oligodendrocyte loss and myelin degradation follow as a consequence of secondary damage. Enhancement of endogenously occurring spontaneous remyelination is critical for SCI repair. In our research, myelin tissue was identified by LFB staining, wherein normal myelin lipids were stained blue but turned white after SCI. Our results showed that GBI treatment strengthened the protective effect of decompression.

The myelin sheath is damaged during SCI, while a large number of neuronal cells undergo apoptosis. Microglia activation and macrophage infiltration also occur in injured tissues, resulting in the exacerbation of SCI [20, 21]. In response to SCI, neurofilaments, which are usually absent in normal cells, are synthesized in large quantities to aid nerve regeneration after SCI [22]. GFAP, a marker of astrocyte damage, is significantly upregulated after SCI, and the markers of activated glial cells, CD11b and CD45, show positive expression [23, 24]. In our study, NF-200, GFAP, CD11b, and CD45 were applied as markers of nerve regeneration, astrocytes, and glial cells. Collectively, the results showed that GBI further downregulated the expression of GFAP and NF-200 after decompression, indicating a significant protective effect. Activated microglia and macrophages produce a large number of inflammatory factors such as NO, TNF-*α*, COX-2, CCLs, and other neurotoxic factors, leading to neuronal death and aggravating nervous system damage. We examined the expression of ten inflammation-related proteins to verify the combined therapeutic effect of GBI and decompression in rats with SCI. Western blot revealed that the combination of decompression and GBI significantly reduced the expression of IL-6, TNF-*α*, CCL1-3, and COX-2, suggesting the effectiveness of combined GBI and decompression therapy against inflammatory factors in vivo. The observations from Nissl and TUNEL staining showed consistent results.

Although GBI has been reported to have neuroprotection and antioxidant activity in different in vitro and in vivo models, it has not been combined with decompression for postspinal injury treatment. Our study validated the effectiveness of combined GBI and decompression therapy in vivo, aiming to provide a reference for the clinical treatment of SCI.

## 5. Conclusions

Based on previous studies that elucidated the molecular mechanism of secondary injury SCI, we found that early surgical decompression significantly ameliorated SCI in rats. Our study employed the classic clinical drug *Ginkgo biloba*, which further reduced inflammatory response after pots-SCI decompression, protected functional cells such as neurons and oligodendrocytes, and inhibited apoptosis to promote SCI repair. As a traditional Chinese medical ingredient, *Ginkgo biloba* can be combined with surgical decompression treatment in clinical practice and contribute to the clinical treatment of SCI.

## Data Availability

The data used to support the findings of this study are available from the corresponding author upon request.

## Supplementary Materials

Supplementary Materials Figure S1: pictures of *Ginkgo biloba* leaves. Figure S2: spinal cord injury model rat.
