# Supplementary figures and images for: Effects of Ginkgo biloba on Early Decompression after Spinal Cord Injury
Source: Evid Based Complement Alternat Med. 2020 Jun 3;2020:6958246. doi: 10.1155/2020/6958246 (PMC7292971; doi:10.1155/2020/6958246)

Graphical Abstract:

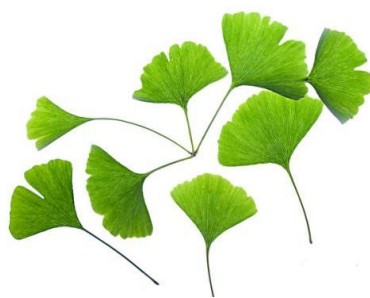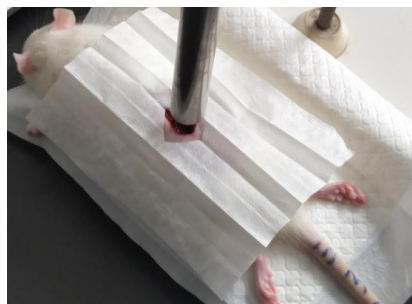

Supplement: Supplementary Materials — Figure S1: pictures of Ginkgo biloba leaves. Figure S2: spinal cord injury model rat. [file 6958246.f1.pdf]
